# Connectomic Analysis of Mitochondria in the Central Brain of Drosophila

**DOI:** 10.1101/2024.04.21.590464

**Published:** 2026-08-07

**Authors:** Kit David Longden, Patricia K Rivlin, Michał Januszewski, Erika Neace, Louis K Scheffer, Christopher Ordish, Jody Clements, Elliott Phillips, Natalie Smith, Satoko Takemura, Lowell Umayam, Claire Walsh, Emily A Yakal, Timothy F Musial, Michael J Devine, Michael B Reiser, Stephen M Plaza, Stuart Berg

## Abstract

Mitochondria are integral to the metabolism and cell biology of a neuron. Electron microscopy images of fly brain volumes, taken for connectomics, can be analyzed for mitochondria as well as the cells and synapses already reported. Here, from the *Drosophila* Hemibrain connectome dataset, we extract, classify, and measure approximately 6 million mitochondria, with the majority located among more than 20 thousand neurons and over 5500 cell types. Each mitochondrion is annotated with its location, orientation, voxel size, and appearance (dark and dense, light and sparse, or medium), and each synapse is linked to its closest mitochondrion. Using these data, we show how the most basic characteristics of mitochondria—volume, distance from synapses, and appearance—vary considerably between cell types and between brain regions. Mitochondria are larger and closer at presynapses than at postsynapses, and presynapses typically have a mitochondrion within one micron. However, cells important for learning and memory, Kenyon cells, have unusually small and sparse mitochondria that are placed with unusual precision near only half of presynapses. We find that mitochondria occupy a greater fraction of cell volume in inhibitory neurons than excitatory cells, dopaminergic neurons have a distinct synaptic positioning of mitochondria, and that glutamatergic neurons have a high fraction of mitochondria with a light appearance. We also find that synapses with more postsynaptic partners have larger presynaptic mitochondria and more distant postsynaptic mitochondria. Finally, we have extended the data model of our public web interface, neuPrint, to record our mitochondria data as searchable subcellular components of neurons in a connectomic database. These results indicate wide-ranging principles for how mitochondrial organization varies by cell type in a dataset of unprecedented size and coverage.

## Full Text Availability

The license terms selected by the author(s) for this preprint version do not permit archiving in PMC. The full text is available from the preprint server.

