# Supplementary material for: Connectomic Analysis of Mitochondria in the Central Brain of Drosophila
Source: bioRxiv. 2026 Jun 30:2024.04.21.590464. Preprint. [Version 3] doi: 10.1101/2024.04.21.590464 (PMC13320868; doi:10.1101/2024.04.21.590464)
Supplement: Supplementary file 1 [file NIHPP2024.04.21.590464v3-supplement-1.pdf]

bioRxiv preprint doi: https://doi.org/10.1101/2024.04.21.590464; this version posted June 30, 2026. The copyright holder for this preprint (which was not certified by peer review) is the author/funder, who has granted bioRxiv a license to display the preprint in perpetuity. It is made available under a CC-BY 4.0 International license.

Supplemental Data

Supplementary Data Table 1: Mitochondrial properties of cells.

Supplementary Data Table 2: Mitochondrial properties of cell types.

Supplementary Data Table 3: Mitochondrial properties of super-ROIs.

Supplementary Data Table 4: Synapse-to-mitochondria distances of cells.

Supplementary Data Table 5: Synapse-to-mitochondria distances of cell types.

Supplementary Data Table 6: Neurotransmitter predictions.

Supplementary Data Table 7: Synapse fan-out of presynapses.

Supplementary Data Table 7: Synapse fan-out of postsynapses.
